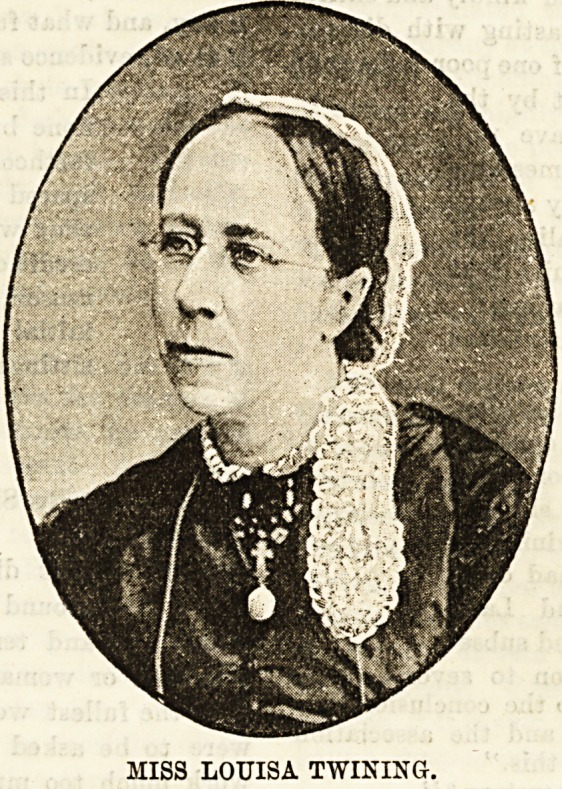# The Workhouse Infirmary Nursing Association

**Published:** 1890-12-06

**Authors:** 


### THE WORKHOUSE INFIRMARY NURSING ASSOCIATION.

### INTERVIEW WITH MISS J. WILSON.

It is a sad and sorrowful world in many of its aspects, but for anyone of a fairly healthy temperament who will take the trouble to inquire a little into the beneficent agencies and activities that are working in its midst, it is impossible to feel anything but exultant hopefulness for its future. On every hand one finds gentle ministries, honest, earnest efforts to mitigate its sorrows and relieve its suffering; in every direction one sees evidences of increasing sympathy, of a developing sense of brotherhood and sisterhood and mutual responsibility.

One of the very best and most significant of these agencies and at the same time one of the most unostentatious, is the Workhouse Infirmary Nursing Association, an organization that may be taken to be officially represented by its honorary secretary, Miss Wilson, a quiet, clear-headed trained nurse, whose keen pleasure in everything pertaining to the ministration for the sick led her into connection with the association of which she is honorary secretary, and respecting which I have come to make a few inquiries.

"Be good enough to tell me something about it, Miss Wilson," began her visitor. "How did it originate? What are its precise objects? What has it done, and what is it hoping to do?"

"Let me answer your last question first," replied Miss Wilson. "What we are hoping to do is to hasten the time when our association shall be no longer necessary. It ought not to be necessary now. If Poor Law Guardians all over the country understood the importance of good nursing, and were sufficiently alive to their duty, there would be no need for our association. To a very large extent, however, they are at present deplorably ignorant and apathetic."

"But how are you endeavouring to educate guardians to a proper sense of their duty? What is the real work of the association?"

MISS LOUISA TWINING.

"The Workhouse Infirmary Nursing Association was formed in order to supply Poor Law Guardians with properly trained nurses. We endeavour to show them how nursing ought to be performed, by sending them well-trained, efficient women, and when they come to understand the difference between proper nursing and that which so commonly prevails in the workhouse infirmaries at the present time, we hope that they will see it to be their duty—simply a part of their business—to provide a proper supply of good nurses for themselves, and without any assistance from such a society as ours."

"But, in the name of goodness, what sort of nurses *do* they get in these places then? Aren't they trained?"

Miss Wilson smiles a little wearily at finding her visitor so efficiently represents the popular ignorance on the subject of sick paupers and their treatment.

"People know so little about these matters," she said. "Occasionally the nurses are simply workhouse inmates, without any training or qualification whatever; or they are *paid* but *untrained* women. If you will take these papers," continued Miss Wilson, handing over some printed matter to her visitor, "you will see something of the state of affairs. It is, of course, in country unions that there is most urgent need of improvement, but even in London it is not an unusual thing to find at the head of a workhouse infirmary a matron who has no competent knowledge of nursing."

Space will not permit of quotations from the printed statements thus submitted, nor is it, perhaps, necessary. Anyone who knows anything of illness may, to some extent, realise the pitiable plight of poor creatures lying on beds of suffering day after day, week after week, away from friends and kindred, with nobody to do the needful services for them but some "wa rdswoman" wholly unqualified for the work, and very possibly full of resentment at having it imposed upon her. Moreover, in country unions every little outlay is often begrudged by the guardians, who look upon the keeping down of the rates as one of the main objects of life. Just imagine, if you can, good reader, what it must be to be "a pauper," dying in a badly equipped workhouse ward, full of pain and weakness and despondency, and to know that you are only one of ninety people—chronic invalids, imbeciles, epileptics—that there is only one nurse for the whole, and during the long, weary night no nurse at all! Was there ever a more pathetic object than "a pauper" struggling out of bed in the dead of night to moisten his lips with water, and then creeping back again to lie down and die, without a soul near him?

"How do you get your nurses, Miss Wilson?"

"As far as our funds permit, we train them ourselves; that is to say, we select young women of thoroughly good character, and send them to some institution where they will be trained in the practical business of nursing for twelve months. We pay their fees, and we require them to enter into an engagement to continue with the association for three years after the expiration of their year of training. We also take women who have been trained apart from our assistance, and who hold a nurse's certificate.

"What can they earn?"

"Twenty to twenty-five pounds a-year, with all found and partial uniform; in country unions, £25 to £30."

"It is not a great income, and the work must be very trying and hard."

"Oh, very; especially in the smaller country infirmaries, where a trained nurse has very often difficulties of the most serious character to contend with. Their work is not understood, and they are often met with every kind of opposition."

"They must have great need of genuine philanthropic principle to sustain them in such work. I am afraid I couldn't endure it."

"Oh, I love nursing," replied Miss Wilson, with quiet fervour. "There is nothing I like so much. One gets so attached to the people when they are ill. Unfortunately I am not strong enough for the work."

The honorary secretary of this association gives you the impression of one with just the endowments of mind and sympathy that are especially required in the best of our hospital nurses and sisterhoods, and it is easy to imagine that she would almost infallibly detect the want of such qualities in those who present themselves to her as probationers.

"And are you really making progress with your work, Miss Wilson?"

"Yes, we are extending very gradually, but we could do much more if we had the funds."

"About what number have you trained?"

"During the ten years the association has been at work we have trained 108 nurses, and we have appointed to situations nearly 300. The applications we have received have been about 350. We have always more applications for nurses than we are able to supply. As we state in our last year's report, we received during the twelvemonth eighty-nine applications from thirty-nine Boards of Guardians, and only fifty-three nurses could be supplied. We want more help, especially from Boards of Guardians. The Local Government Board readily consent to guardians subscribing to our association; but there are only about twenty-one that do so throughout the country."

This is not altogether creditable to guardians, for the work that the Workhouse Infirmary Nursing Association is doing is one that commends itself to its common-sense, no less than to one's right feeling, and no institution of the kind can be worked much more economically than this. It has never had on an average an income of more than £250 a-year, and probably no £250 a-year spent was more fruitful in blessing to the neediest of suffering humanity than that which has gone year after year in providing them with kindly and skilful attention when broken in health and wasting with disease. There was infinite pathos in the words of one poor sufferer to a kindly and competent nurse sent out by the association: "Don't go away and leave us," pleaded the poor mortal; "if anyone comes here with a little feeling in their hearts, they always leave directly." Which of us can realise the dreary experiences of heart-broken anguish that probably wrung out that cry from the patient?

"You haven't told me how your association originated, Miss Wilson."

"It originated with the Marchioness of Lothian, who had been a Poor Law Guardian and constant visitor to a large London infirmary connected with one of the workhouses. In 1879 she and Lady Montagu invited Miss Louisa Twining to meet them and talk over matters that had come under her notice. Miss Twining and Lady Lothian visited the infirmary together, and subsequently paid similar visits of inspection to several other institutions of the kind. They came to the conclusion that there was great room for improvement, and the association was formed for the purpose of effecting this."

"And you were appointed honorary secretary?"

"No; my friend Miss Twining was first honorary secretary. I joined the association in 1880."

Honorary secretaries are not perhaps always the best of secretaries. But by very general consent it seems to be allowed that the Workhouse Infirmary Nursing Association is very fortunate in having an exceptionally able and earnest officer in Miss Wilson, who, as all who know her will regret to learn, is suffering from what it is to be feared will prove chronic writers' cramp. There can be little doubt that the arduous work she had done for the association for many years past has contributed in no slight degree to a malady which has incapacitated her right hand. Nevertheless she is still zealously endeavouring to extend the usefulness of this invaluable organisation. She would be glad if the Local Government Board could be persuaded to put upon guardians what pressure may be practicable to induce them to employ only properly trained nurses; and she would be delighted to receive subscriptions either from Boards of Guardians who do not at present subscribe, or from anyone with sufficient sympathy with the extremely poor in their sickness and suffering to desire that they shall have what alleviation of their affliction competent nursing can do for them. Two hundred and fifty pounds a year is not a tithe of what ought to be forthcoming for so eminently humane and necessary a purpose. All who would like to help should communicate with Miss Wilson at the office of the Association, 6, Adam Street, Adelphi.

## Figures and Tables

**Figure f1:**